# Factors of Influence on the Performance of a Short-Latency Non-Invasive Brain Switch: Evidence in Healthy Individuals and Implication for Motor Function Rehabilitation

**DOI:** 10.3389/fnins.2015.00527

**Published:** 2016-01-21

**Authors:** Ren Xu, Ning Jiang, Natalie Mrachacz-Kersting, Kim Dremstrup, Dario Farina

**Affiliations:** ^1^Department of Neurorehabilitation Engineering, Bernstein Center for Computational Neuroscience, University Medical CenterGöttingen, Germany; ^2^Institute of Computer Science, Faculty of Mathematics and Computer Secience, Georg-August UniversityGöttingen, Germany; ^3^Department of Systems Design Engineering, University of WaterlooWaterloo, ON, Canada; ^4^Center for Sensory-Motor Interaction, Department of Health Science and Technology, Aalborg UniversityAalborg, Denmark

**Keywords:** brain-computer interface, motor intention detection, ballistic and repetitive task, movement-related cortical potential, sensory-motor rhythm

## Abstract

Brain-computer interfacing (BCI) has recently been applied as a rehabilitation approach for patients with motor disorders, such as stroke. In these closed-loop applications, a brain switch detects the motor intention from brain signals, e.g., scalp EEG, and triggers a neuroprosthetic device, either to deliver sensory feedback or to mimic real movements, thus re-establishing the compromised sensory-motor control loop and promoting neural plasticity. In this context, single trial detection of motor intention with short latency is a prerequisite. The performance of the event detection from EEG recordings is mainly determined by three factors: the type of motor imagery (e.g., repetitive, ballistic), the frequency band (or signal modality) used for discrimination (e.g., alpha, beta, gamma, and MRCP, i.e., movement-related cortical potential), and the processing technique (e.g., time-series analysis, sub-band power estimation). In this study, we investigated single trial EEG traces during movement imagination on healthy individuals, and provided a comprehensive analysis of the performance of a short-latency brain switch when varying these three factors. The morphological investigation showed a cross-subject consistency of a prolonged negative phase in MRCP, and a delayed beta rebound in sensory-motor rhythms during repetitive tasks. The detection performance had the greatest accuracy when using ballistic MRCP with time-series analysis. In this case, the true positive rate (TPR) was ~70% for a detection latency of ~200 ms. The results presented here are of practical relevance for designing BCI systems for motor function rehabilitation.

## Introduction

In the past decade, non-invasive brain-computer interfacing (BCI) based assistive technology has been proposed as a novel rehabilitation tool for people suffering of motor disorders (Daly and Wolpaw, 2008; Shih et al., 2012), such as stroke (Ramos-Murguialday et al., 2013) and spinal cord injury (Enzinger et al., 2008). In BCI systems for neurorehabilitation, the volition of subjects is detected from brain signals. Such a brain switch is used to control a neuroprosthetic device, such as an electrical stimulator (Niazi et al., 2012; King et al., 2014) or a robotic system (Xu et al., 2014b), to close the sensory-motor loop for either restoring motor function or modulating neural pathways (Shih et al., 2012).

As the first step of these closed-loop systems, accurate online detection of motor intention is a crucial and challenging task for non-invasive neural recordings such as scalp electroencephalography (EEG), mainly due to its low spatial resolution and poor signal-to-noise ratio. For neuroprosthesis control in general, the acceptable delay between intention and action is ~200 ms (Lauer et al., 2000). In particular, the detection latency is crucial in inducing Hebbian associative neural plasticity for rehabilitation purposes (Hebb, 1949). The efficiency of plasticity induction would be extremely slow, if at all possible, when the artificially afferent triggered by the brain switch arrived at the cortical level either too early or too late relative to motor intention (Mrachacz-Kersting et al., 2012).

Several signal processing approaches have been proposed for motor intention detection from EEG (Venkatakrishnan et al., 2014). Among these methods, two main EEG signal modalities have been explored for the purpose of motor rehabilitation: sensory motor rhythms (SMRs; Yuan and He, 2014) and movement related cortical potentials (MRCPs; Xu et al., 2014a), a type of slow cortical potential.

SMR corresponds to an increase or decrease in power at various subbands of EEG signals recorded over the sensory motor cortex (e.g., *Cz* for foot movements), prior to, during and after movement, or movement imagination (Yuan and He, 2014). The increase of subband power implies that the neurons in the corresponding cortical area discharge more synchronously than at baseline and therefore it is referred to as event-related synchronization (ERS; Pfurtscheller and Lopes Da Silva, 1999). Conversely, the decrease of subband power corresponds to less synchronous neural activity, termed event-related desynchronization (ERD; Pfurtscheller and Lopes Da Silva, 1999). Immediately after movement imagination, an ERS is usually observed in the beta-band (~20 Hz; Pfurtscheller and Solis-Escalante, 2009). This is also referred to as beta-rebound. In most SMR-based BCI studies for neurorehabilitation, the movement imagery that the subjects were instructed to perform was repetitive movement, such as foot tapping, with a few exceptions (Pfurtscheller and Solis-Escalante, 2009).

MRCP is another EEG signal modality observable on the sensory motor cortex prior to, during and after movement or movement imagery. It is characterized by a slow negative deflection of the near-DC component in the EEG signal before movement or movement imagery, reaching its peak of negativity near the onset of movement or movement imagination, and followed by a positive rebound before the signal returns back to its reference level (Jahanshahi and Hallett, 2003). MRCP is characterized by a time-series change at a very narrow low frequency content (0.05–3 Hz; Jahanshahi and Hallett, 2003). In MRCP-based BCI studies, the movement or imagined movement is usually executed once, often as a brisk or ballistic task, as opposed to the repetitive movement used in SMR-based studies. More recently, a combined approach of subband SMR and time-series MRCP has been proposed (Ibáñez et al., 2014). This fusion approach yielded improved performance for the detection of ballistic reaching movement (Ibáñez et al., 2014).

Due to the low signal-to-noise ratio of EEG, spatial filtering is usually used as a pre-processing step to enhance the desired feature. Among them, common spatial pattern (CSP) has been very successful in processing SMRs (Ang et al., 2008; Blankertz et al., 2008), particularly when the channels are more than 20. When the channels are less (typically <10), the Laplacian filter has been widely used for both signal modalities (Müller-Putz and Kaiser, 2010; Niazi et al., 2011).

The above survey indicates that a motor imagery based short-latency brain switch is predominantly influenced by three factors: the type of motor task (ballistic or repetitive), the frequency band (e.g., MRCP, alpha or beta band) of EEG, and the corresponding processing technique (subband power estimation or time-series analysis). In previous studies, the effect of some of these factors was partly investigated, e.g., SMR in brief and sustained movement (Cassim et al., 2000; Alegre et al., 2003) or MRCP vs. SMR in real movements (Toro et al., 1994; Babiloni et al., 1999; Filipovic et al., 2001). More recently, there have been studies on the analyses of the frequency band in motor intention detection (Garipelli et al., 2013; Ibáñez et al., 2014; López-Larraz et al., 2014). However, to date, there has been no direct comparison of advantages and disadvantages of all the above factors in the context of a short-latency brain switch for rehabilitation purposes. In this study on healthy subjects, these factors are directly compared in their influence on the low latency detection of movement intention, in an attempt to provide a guideline for BCI researchers working toward closed-loop neuroprosthetic applications.

## Methods

### Subjects

Ten healthy volunteers (seven male and three female, age: 26.5 ± 4.6 years) participated in the study. Informed consent was obtained from all participants, and ethical approval was provided by the local ethics committee in accordance with the Declaration of Helsinki.

### Experimental setup

• EEG

Nine channels of EEG were acquired with an active electrode system (ActiCap, Brain Products, Germany) and 16-channel EEG amplifier (g.USBamp, gTec GmbH, Austria). The electrodes were placed in the standard 10–20 locations at Cz, Fz, F3, F4, C3, C4, P3, P4, and Pz. The ground and reference located on AFz and the left earlobe, respectively. Sampling frequency was 1200 Hz, with no hardware filter. During all experiments, the impedances of all channels were monitored regularly to ensure that they were below the recommended values indicated by the manufacturers of the active electrode system.

• EMG

Surface electromyography (EMG) signals were collected from the tibialis anterior (TA) muscle of the right foot with disposable electrodes (Neuroline 720, Ambu). EMG was acquired by the last channel of the g.USBamp amplifier, with separate ground and reference electrode from the EEG channels. A monopolar electrode was placed on the mid-belly of the right TA muscle while the reference and ground electrodes were placed on the bony surface of the right knee and right ankle, respectively.

### Experimental procedure

**Figure 1:**
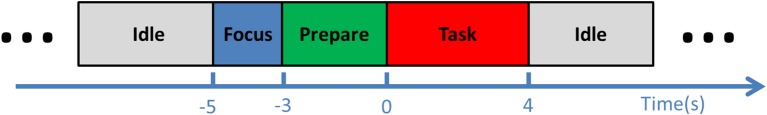
**Experimental procedure**. Each trial began with an idle phase, followed by a 2-s focus phase and a 3-s preparation phase. In the consequential task phase, the subject was instructed to perform ballistic or repetitive imagination of dorsiflexion.

During an experimental session, the subject was comfortably seated in a chair, ~1 m from a computer screen. Participants were instructed to look at the center of the screen and to follow the cue presented, minimizing eye movements. During the experiment, the cue on the screen indicated four states (Figure 1): idle, focus, preparation, and task. Each trial started from the 5-s idle phase, during which the subjects could adjust their position as they wished. In the second phase, the subjects were asked to focus on the screen without moving. This was followed by the preparation phase, where the subjects were instructed to follow the 3-s backwards counting presented on the screen, and to start imagining the movement immediately when it turned to the task phase, which lasted for 4 s. One trial ended with the next idle phase before the next trial commenced.

Each experimental session was divided into six runs, which consisted of three ballistic and three repetitive runs. The ballistic and repetitive runs were identical, except in the task phase. For ballistic runs, the subjects were instructed to imagine performing ballistic dorsiflexions at the beginning of the 4-s task phase; in repetitive runs, they were continuously repeating motor imagery for the whole 4-s task phase. The subjects were instructed to perform the repetitive task at a moderate speed, i.e., around once per second. The TA muscle activity was monitored through the EMG recording, and those trials with visible EMG signal were not included in further analysis. Each run comprised approximately 20 trials of ballistic or repetitive imaginary movements. The duration of each run was ~6 min. The order of ballistic and repetitive runs was randomized.

### EEG processing algorithm

#### MRCP and SMR morphology analysis

The nine channels of EEG were band-pass filtered (2nd order Butterworth) at 0.05–3 Hz for MRCP analysis, (Xu et al., 2014a) and 4–40 Hz for SMR analysis (Planelles et al., 2014). Then a large Laplacian spatial filter centered at *Cz* (see Equation 1) was used to enhance the signal-to-noise ratio of the “virtual” *Cz* channel, which was then processed in subsequent steps.

(1) $$\begin{array}{l} virtual\text{\_}Cz=Cz\;-\sum_{i}{CH}_{i}∕8 \end{array}$$

Where *CH*_*i*_ stands for the eight channels around *Cz*.

In subsequent data segmentation, the data from *t* = −3 to *t* = 6 s, w.r.t. the task onset, of the filtered virtual *Cz* were extracted for each trial. For both SMR and MRCP, the reference interval, from which the baseline value was calculated, was −3 to −2 s (3 to 2 s before motor imagery onset).

1. MRCP morphology analysis
  For each subject and each movement type, a statistical comparison was performed on the characteristics of the morphorlogy of the MRCP (see details in the Section of Statistical Methods below).
2. SMR morphology analysis
  The power spectral density (PSD) of each trial was calculated over 1 s windows overlapped for 0.5 s using Hamming windows (Matlab function pwelch). For each subject and each movement type, a Bootstrap test was performed between the PSD of the SMR at each time-frequency point and the reference PSD of the SMR in a baseline window. The time-frequency SMR characterization was quantified as follows (Pfurtscheller and Lopes Da Silva, 1999):
  (2) $$\begin{array}{l} {SMR}_{f,t}\%=\frac{A_{f,t}\;-\;R_{f}}{R_{f}} \end{array}$$
  where *SMR*_*f, t*_% is the relative power of the SMR at time *t* and frequency *f, A*_*f, t*_ is the absolute power of the SMR at the same time-frequency point, and *R*_*f*_ is the power of the reference interval for the frequency f. A positive *SMR*_*f, t*_% value indicates an ERS, while a negative value of SMR indicates an ERD.

#### Time-series feature extraction and motor imagery detection

In order to analyze the information content in EEG for motor intention detection, six types of time-series features were extracted with band-pass filters at MRCP (0.05–3 Hz), Theta (4–7 Hz), Alpha (8–15 Hz), Beta (16–30 Hz), lower Gamma (31–40), and the full frequency band (0.05–40 Hz), followed by a large Laplacian filter centered at *Cz*. In order to evaluate the BCI performance, a three-fold cross-validation was used, in which two runs of the virtual *Cz* (either ballistic or repetitive) were taken as the training set, while the remaining ballistic or repetitive run was used as the testing set. For the training set, the virtual *Cz* was segmented into portions of 2-s segments with 0.1-s increments. The segments between −1 to 1 s with respect to the task onset were labeled as “signal” portion, while all the remaining segments were labeled as “noise” portion. The testing set was treated in a pseudo-online way, mimicking the real online processing, where the data arrives continuously with a 2 s-length window and a refresh step of 0.1 s.

For the time-series feature, a manifold-learning based method called Locality Preserving Projection (LPP; He and Niyogi, 2003), followed by a Linear Discriminant Analysis (LDA) classifier was trained and used for detection. LPP-LDA has previously been implemented for MRCP detection with good performance (Xu et al., 2014a). The procedure is described briefly here. The “signal” and “noise” portions were first projected to a lower dimension using LPP, which maintained the intrinsic structure in the original manifold with high dimension (He and Niyogi, 2003). A LDA classifier was trained using the LPP projected training data. Once the LPP-LDA classifier was trained, the testing segments were projected by the obtained LPP projection, and the trained LDA was used to classify the testing data into either “signal” or “noise.” A detection of motor intention would be registered when a number of continuous windows [referred to window number (WN)] were classified as “signal.” One detection would be determined either as a true or false detection according to the detection latency (DL), i.e., the time difference between the detection and the task cue. If the DL was between -1 and 1 s, the detection was considered a true detection, otherwise a false detection. It should be emphasized here that the target of this study is a short-latency brain switch. Therefore, we only considered these detections within a few hundreds microseconds, even though signals outside this range may improve the accuracy for modalities such as Beta rebound. The true positive rate (TPR), false positive (FP) per minute, and DL were calculated to quantify the BCI performance. Compared with false positive rate (or specificity) which is generally used for evaluating binary classification (Hashimoto and Ushiba, 2013; Jochumsen et al., 2013) FP/min is more suitable for quantifying the performance of continuous detection in a (pseudo-) online paradigm, as was done in Niazi et al. (2011) and Xu et al. (2014a). As any detector, there is a trade-off between TPR and FP. Both TPR and FP are constrained by WN, whose increment would make the detection stricter (more difficult), leading to lower TPR and smaller FP. In order to objectively compare the BCI performance, we chose the WN value for which the FP was smaller than or equal to 8/min for all comparisons. Thus, only the TPR and DL were statistically compared.

#### Subband power estimation and motor imagery detection

The subband power was also used as a direct feature for classification. For this purpose, the powers of the virtual *Cz* channel at the six frequency bands were estimated using the Welch periodogram with a resolution of 1 Hz. As for the time-series features, the window duration was of 2 s, with increments of 0.1 s. “Signal” and “noise” portions were the same as for the time-series features, and the same three-fold cross-validation method was used to test performance.

Since the dimensions of the subband power features are small, no dimension reduction method was used. The “signal” and “noise” portions were directly used to train a LDA classifier, which was used for detection of motor intention from the testing set. All BCI performance criteria were calculated with the same steps as in the time-domain processing, and analyzed using the statistical methods described in the following.

### Statistical methods

A paired *t*-test was performed for MRCP morphology analysis. The comparison was between the amplitude of the MRCP for the reference interval (i.e., mean value of −3 to −2 s w.r.t. task onset) and the magnitude of each 0.1 s-length segments of the virtual *Cz* outside the reference interval. A Holm-Bonferroni correction was performed for this multiple comparison, and the significance level was set to 0.05.

Three-way repeated ANOVA was used to investigate the effect of the three factors on BCI performance. The independent variables were TPR and DL. The three main factors were motor task (ballistic and repetitive imagination), frequency band (MRCP, Theta, Alpha, Beta, Gamma, and full frequency bands), and processing technique (time-series analysis and subband power estimation). A full model ANOVA with all interaction terms was performed first and, when significant interactions were detected, *post-hoc* tests (Tukey simultaneous test with significance level of 0.05) were performed.

## Results

### Signal morphology

#### MRCP morphology

**Figure 2:**
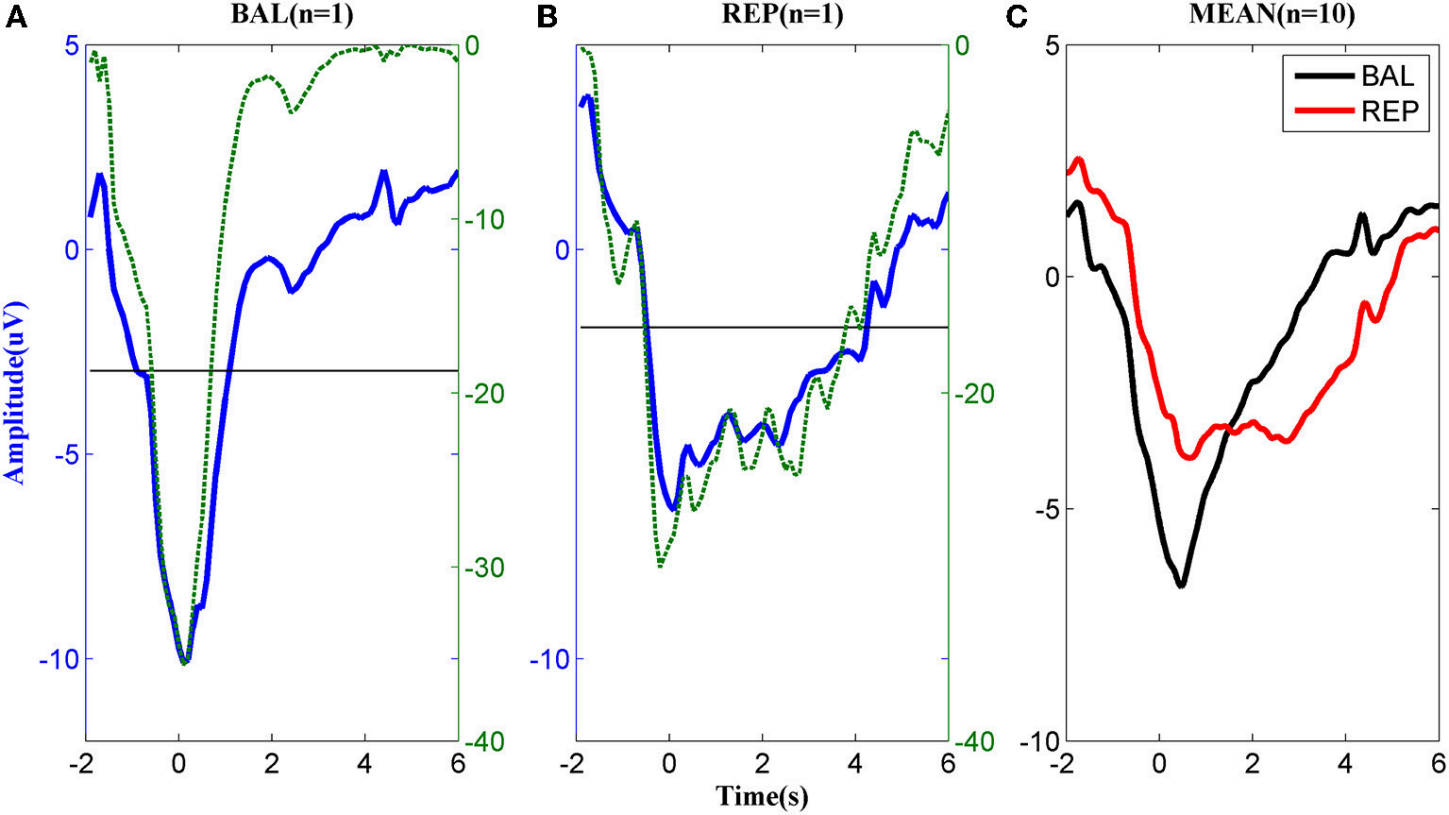
**MRCP corresponding to (A) ballistic (BAL) and (B) repetitive (REP) motor imagery from a typical subject**. The dashed lines indicate the logarithmic *p*-value of the paired *t*-test between the MRCP (either ballistic or repetitive) and its reference between −3 and −2 s., while the solid horizontal line indicates significance level. **(C)**: The average MRCP over the 10 subjects. The black line corresponds to ballistic imagery and the red line to the repetitive motor imagery.

The MRCPs of a typical subject performing the two types of motor imageries are shown in Figures 2A,B. For the ballistic task (Figure 2A), the MRCP started to decrease approximately 2 s prior to the task onset, reached the negative peak around *t* = 0 s, and returned to the baseline in approximately 2 s after the task onset. In the repetitive task, the MRCP (Figure 2B) had a similar shape to that of the ballistic case before *t* = 0 s but the rebound phase was much longer in duration. Before task onset, both the ballistic and repetitive MRCP showed significant differences to the baseline starting from −1 s. However, the characteristics of the two motor tasks were different for the rebound part. No significant difference was found between the ballistic MRCP and the baseline from the time of 1 s, indicating it already returned back to the baseline. On the other hand, the repetitive MRCP still showed significance as late as 4 s after the task onset.

The above difference between ballistic and repetitive tasks was consistent across all subjects, as shown in Figure 2C. These results indicate a strong predictive power of MRCPs in detection of movement intention of the subjects for both types of motor imageries.

#### SMR mapping

**Figure 3:**
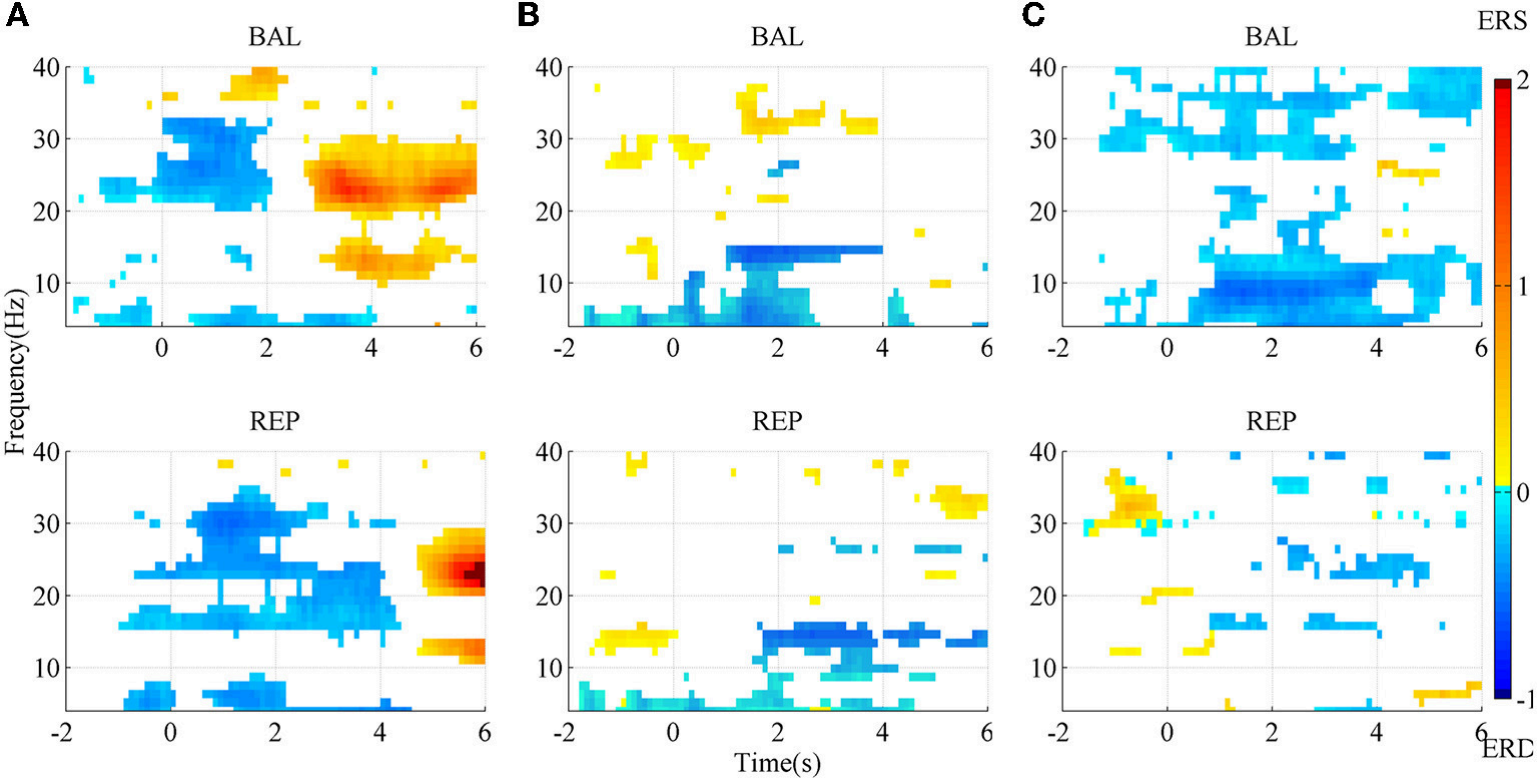
**SMR mapping from three representative subjects**. **(A)** subject A; **(B)** subject B; **(C)** subject C. BAL and REP stand for ballistic and repetitive task, respectively. Only those points with significance in bootstrap test are presented. Red area indicates ERS, while blue area is ERD.

The SMR maps for three representative subjects for the two types of movement imageries are shown in Figure 3. For subject A, there was an evident ERD starting slightly earlier than the task onset, between the Beta and the lower Gamma band (above ~20 Hz) for the ballistic imagery, while it corresponded to a larger bandwidth for repetitive imagery. There was also an evident ERS in the Alpha and Beta bands for both tasks, but the repetitive ERS occurred much later than the ballistic one. However, the SMR landscape was very different for subject B, whose ERD and ERS mainly appeared in the Alpha and lower Gamma band, respectively. Subject C showed still other characteristics. The ERD occurred over almost the full band for the ballistic imagery, whereas it did not present a clear pattern in the repetitive imagery. Moreover, for all subjects, both the ERD and ERS occurred earlier in case of ballistic imagery with respect to repetitive imagery. ERS appeared before imagery onset in both ballistic and repetitive tasks of subject B, and also in the repetitive task of subject C.

In summary, the SMR mapping differed substantially among the subjects, thus a general average across the subjects would not be meaningful and therefore is not reported.

### BCI performance

**Figure 4:**
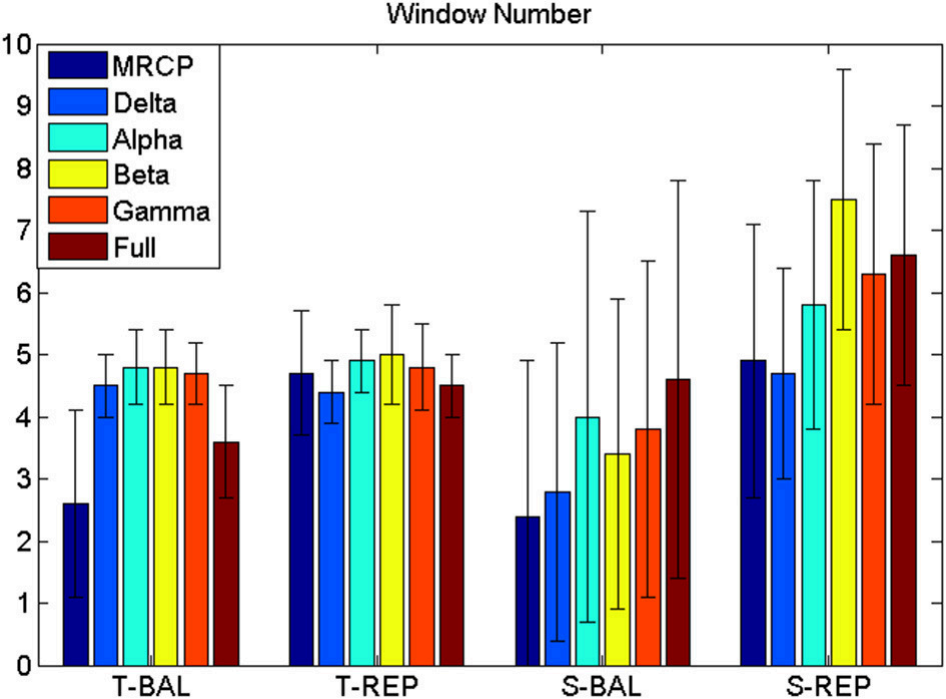
**Window number (WN)**. WN is chosen where false positive ≤ 8 min^−1^. T-BAL and T-REP represent ballistic and repetitive task with time serial analysis, while S-BAL and S-REP stand for these two motor task with subband power estimation.

The BCI performance in detection of motor imagery is summarized in Table 1. The WN is shown in Figure 4. The ballistic MRCP with time-series analysis reached the highest TPR (70 ± 20%), followed by ballistic Beta band with subband power estimation (57 ± 19%). SMRs with time-series analysis yielded shorter DL (<100 ms), however the corresponding detection accuracy was extremely low (~30%). For each frequency band of either motor task (except the full band of the ballistic task), the time-domain technique resulted in shorter DL than the frequency-domain technique.

**Table 1:** **BCI performance**.

**Frequency band**	**Time-series analysis**				**Subband power estimation**			
	**BAL**		**REP**		**BAL**		**REP**	
	**TPR%**	**DL/ms**	**TPR%**	**DL/ms**	**TPR%**	**DL/ms**	**TPR%**	**DL/ms**
MRCP	**70±20**	267±121	44±13	197±201	47±18	389±113	47±25	239±228
Theta	41±8	64±151	41±9	132±127	47±21	369±128	43±20	204±230
Alpha	32±8	37±138	28±8	83±180	48±18	260±218	48±16	230±176
Beta	32±10	106±144	32±9	36±113	57±19	282±198	50±16	142±243
Gamma	37±8	40±97	36±10	37±161	53±16	256±121	48±16	210±111
Full	54±18	224±108	30±7	185±202	51±19	192±195	46±11	254±174

*BAL and REP stand for ballistic and repetitive imagery, respectively. True positive rate (TPR) is the ratio between true detection and the total number of trials in the testing set. Detection latency (DL) is the timing difference between the detection point and the task onset. The TPR and DL were chosen where false positive ≤8 min−1. The positive DL indicates that the detection happened after the task onset. The best accuracy is indicated in bold*.

**Figure 5:**
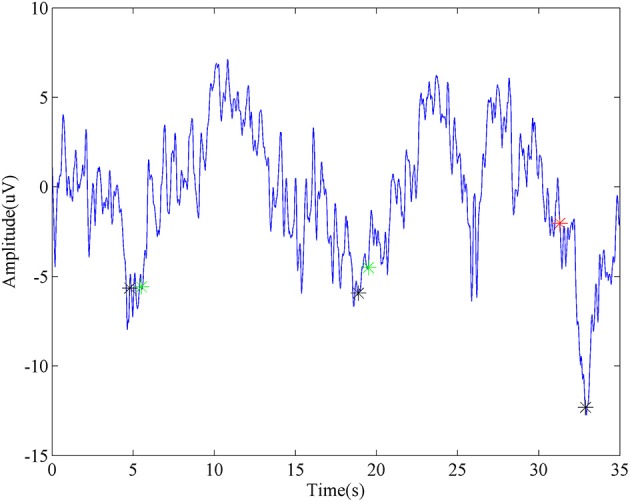
**Representative detection of MRCP**. The blue line is a segment of virtual *Cz* from ballistic task. Black stars stand for the task onsets. Green stars represent true detections, while the red star is a false detection.

A representative segment of detecting ballistic MRCP with time-serial analysis was shown in Figure 5. Three black stars stand for the onsets of three consecutive tasks. For the first two, the detector successfully identifies them, which were labeled as Green stars. Moreover, this method demonstrated its robustness again moderate variation which appeared after the second trial (~25 s). In spite of this, there is still a false detection (labeled as a red star) when huge noise was introduced during the third trial.

#### Analysis of true positive rate

For a meaningful comparison between methods, the TPR is reported in all cases for the same level of false positives (≤8 min−1). The Three-way ANOVA on TPR found no three-way interaction (*p* = 0.074). Neither was the interaction between motor task and processing technique (*p* = 0.283). However, there was a significant interaction between motor task and frequency band (*p* = 0.015), as well as between processing technique and frequency band (*p* < 0.001). Therefore, we performed *post-hoc* tests on the significant interactions.

Focusing on the interaction between motor task and frequency band, the post-hoc comparison revealed that the MRCP of the ballistic task yielded the highest TPR (60 ± 22%). This was comparable to the full band of the same task, and significantly better than all other combinations (no significance was found among them). In addition, for both the MRCP and the full band, the ballistic task significantly outperformed the repetitive task.

For the other significant interaction between processing technique and frequency band, the difference depended on each factor. MRCP with time-series analysis provided the highest TPR (57 ± 22%), which was significantly better than all other frequency bands with the same processing technique. For the other processing technique, i.e., subband power analysis, there was no significant difference among frequency bands. Furthermore, it was observed that, for Alpha and Beta band, subband power analysis outperformed time-series analysis.

#### Analysis of detection delay

There was no three-factor interaction (*p* = 0.451), nor two-factor interactions (*p* = 0.197, 0.532, 0.081). Both the processing technique and frequency band were significantly different (*p* < 0.001 and *p* = 0.004, respectively). The *post-hoc* comparison revealed that the time-domain technique had lower DL than the frequency-domain technique (117 ± 169 vs. 252 ± 194 ms), while the MRCP resulted in longer DL than Alpha, Beta, and Gamma band (265 ± 185 vs. 145 ± 205, 129 ± 193, and 125 ± 159 ms, respectively).

#### Summary

Based on the above statistical analysis of TPR and DL, we summarize the influence of the three factors here. The ballistic task, is preferable over the repetitive task, as it yielded higher TPR for both MRCP and full band. Even though MRCP's DL was slightly larger than for some SMRs, MRCP was still the best choice among all six frequency bands, given its highest TPR for both tasks and for the time-series processing technique. Time-series analysis outperformed subband power analysis, mainly due to a significantly shorter DL.

In summary, this comprehensive comparison on motor intention detection with two motor tasks (ballistic and repetitive), six frequency bands (MRCP, Theta, Alpha, Beta, Gamma, and full band), and two processing techniques (time-series analysis and subband power estimation) showed that the combination of ballistic, MRCP and time-series analysis significantly is preferred among all the considered options.

## Discussion

As a crucial aspect of closed-loop rehabilitation systems, the detection of the motor intention from scalp EEG is a central challenge in motor imagery based BCI. In recent years, there has been an increasing number of publications of clinical studies using SMR-based BCI (Ramos-Murguialday et al., 2013; Ang et al., 2014a,b; Li et al., 2014; Mukaino et al., 2014; Ono et al., 2014). On the other hand, MRCPs have also been proven as a promising signal type, particularly for neuromodulation purposes due to its short detection latency (Mrachacz-Kersting et al., 2012; Niazi et al., 2012; Xu et al., 2014b). Preliminary studies of MRCP-based BCI applied to chronic stroke patients have also been reported (Mrachacz-Kersting et al., 2015).

According to the Hebbian principle of associative plasticity (Hebb, 1949), neuroplasticity would only be induced when the motor intention and the task specific afferent feedback, e.g., passive movement delivered by an orthosis, occur synchronously in a cause-and-effect fashion. Therefore, an effective neuromodulation system requires not only accurate algorithms, but also algorithms that present short detection latencies, ideally shorter than 300 ms.

In the past decades, SMR has been the main signal modality used for detection purposes. TPRs above 80% were reported with Beta ERS (Müller-Putz and Kaiser, 2010; Wang et al., 2012), while the performance of ERD was also demonstrated to be above 70% (Planelles et al., 2014; Yang et al., 2014). However, the issue of detection latency was largely overlooked in these studies, with only one exception which reported a latency in the range of seconds (Hashimoto and Ushiba, 2013). In this study, the best TPR using SMR was 57%, for the beta band in the ballistic task using subband power estimation. Compared with previous studies, the performance here decreased obviously, mainly due to the limited range of latency. In these previous studies using SMR, detection latency was rarely reported. Its average latency can be more than one second if we take into consideration those detections which occur several seconds after the task onset (Hashimoto and Ushiba, 2013). On the contrary, in the current study, short latency detection is essential, as it is mandatory for the purpose of plasticity induction (Mrachacz-Kersting et al., 2012). As such, those detections occurring after 1 s were counted as false detections rather than true ones, resulting in a latency of several hundred milliseconds as shown in Table 1. Taking later windows into consideration would likely improve TPR, but would lead to a long-latency brain switch, which would be out of the focus of this study.

On the other hand, slow cortical potentials, such as MRCP, have been investigated for movement intention detection in recent years (Qian et al., 2010; Bai et al., 2011; Niazi et al., 2011; Lew et al., 2012; Bulea et al., 2013, 2014; Bhagat et al., 2014; Xu et al., 2014a). In the current study, the average TPR and DL was 70% and <300 ms for motor imagination, consistent with the results reported in previous studies (Niazi et al., 2011, 2012; Xu et al., 2014a,b). The relatively good performance in TPR (>70%) is essential for the high efficiency of MRCP-based BCI system. In particular, the detection latency of a few hundred milliseconds was demonstrated to be crucial for plasticity induction (Mrachacz-Kersting et al., 2012).

In this study, we demonstrated that ballistic motor imagery task, frequency band of MRCP, and time-series analysis is the optimal combination in terms of detection performance. Other options investigated are sub-optimal, mainly due to a trade-off between TPR and DL.

We also observed that repetitive SMR with subband power estimation was significantly better in accuracy than the ballistic one. This is in accordance with the discussion by Pfurtscheller & Solis-Escalante that SMR in repetitive task is easier to classify (Pfurtscheller and Solis-Escalante, 2009).

The different EEG features between the ballistic and repetitive task may be attributed to the difference in their afferent input (Cassim et al., 2000). As explained by Bear (Bear et al., 2007), compared to the repetitive task which has sufficient time for the sensory-motor loop to feedback, the ballistic movement, once initialized, is too fast to adjust. In addition, this difference may also be explain by the fact that there is an inhibitory process immediate following the ballistic task (Alegre et al., 2003), while this is not the case for the repetitive task.

The morphological difference between ballistic and repetitive tasks may mainly contribute to the differences in the detection performance of the two motor tasks. The prolonged negative phase in repetitive MRCP (in Figure 2) makes its rebound feature after task onset not as distinct as the ballistic one. That is why the accuracy of repetitive MRCP with time-series analysis is much lower than the ballistic one. On the other hand, in the case of subband power analysis, the frequency band did not have a significant influence on accuracy. This is likely explained due to the observed variability in the optimal frequency bands among individual subjects (see Figure 3).

### Limitations

The above comparison was performed only on healthy subjects. Previous findings mostly support the similarity in slow EEG waves (e.g., MRCP) between healthy and stroke or spinal cord injured patients (Castro et al., 2007; Mattia et al., 2009; Niazi et al., 2011; Xu et al., 2014c), despite their non-negligibly distinct features such as onset and amplitude (Yilmaz et al., 2014), whereas SMRs trend to have greater difference on patients with central neural injury (Tran et al., 2004; Gourab and Schmit, 2010; Müller-Putz et al., 2014). Other different factors such as medication and mental status in patients could be challenging for clinical measurements. Therefore, further investigation on the target patient population is necessary.

In addition, the combination of several features with different processing techniques, such as those presented in Ibáñez et al. (2014) and López-Larraz et al. (2014), was not investigated in the current study. This study focused exclusively on a general comparison without subject-specific optimization, the combined features, e.g., SMR and MRCP, would be worthy to investigate in future work.

## Conclusion

In this study, we performed a comprehensive comparison of motor task, frequency band, and processing technique, to investigate their influence on the performance of a short-latency brain switch. The morphological investigation found cross-subject consistency in MRCP, which supports its advantage for a subject-independent use. The BCI detection performance was maximized by using the ballistic imagery task, the DC bandwidth (MRCP), and the time-series analysis.

## Author contributions

Conceived and designed the experiments: RX, NJ, NM, KD, DF. Performed the experiments: RX, NJ. Analyzed the data: RX, NJ, DF. Wrote the paper: RX, NJ, NM, DF.

### Conflict of interest statement

The authors declare that the research was conducted in the absence of any commercial or financial relationships that could be construed as a potential conflict of interest.
